# Extraction d'un corps étranger trachéo bronchique à l'aide d'un uretheroscope

**DOI:** 10.11604/pamj.2015.20.74.6002

**Published:** 2015-01-28

**Authors:** Hicham Kechna, Omar Ouzzad, Youness Aissaoui, Karim Nadour, Rachid Zaini

**Affiliations:** 1 Service Anesthésie Réanimation, Hôpital Militaire, Guelmim, Maroc; 2 Service oto-rhino-laryngologie, Hôpital Militaire, Guelmim, Maroc; 3 Service Urologie, Hôpital Militaire, Guelmim, Maroc

**Keywords:** Corps étranger intratrachéobronchique, extraction, fibroscopie, tracheobronchial foreign body, extraction, fibroscopy

## Abstract

Les corps étrangers intratrachéobronchiques (CEITB) sont des accidents fréquents chez les enfants. Dans les pays développés, l'extraction de ces CEITB est réalisée grâce à la fibroscopie bronchique ou à la bronchoscopie rigide. Le recours à la chirurgie est rare. Dans notre contexte, le plateau technique adéquat est inexistant. Des alternatives d'extraction s'imposent afin d’éviter l’évacuation sanitaire, pas toujours à la portée des patients, mais surtout pour faire face à une mort imminente tel est le cas dans notre observation. Nous décrivons l'extraction d'un corps étranger radio-opaque trachéobronchique responsable d'une hypoxie sévère à l'aide d'un uréteroscope.

## Introduction

L'inhalation d'un corps étranger de l'arbre trachéo-bronchique (CEATB) est une cause fréquente de détresse respiratoire chez l'enfant. Elle peut être responsable d'un tableau d'asphyxie aiguë, pouvant être à l'origine du décès si des manoeuvres d'extraction ne sont pas rapidement réalisées [1, 2].

L'extraction des CEATB est relativement aisée dans les pays médicalisés avec l'essor technologique de l'endoscopie bronchique et ses accessoires. La bronchoscopie rigide reste l'outil de base pour extraire les CEATB de l'enfant en dépit des progrès de la vidéofibroscopie bronchique [3]. Dans les pays en développement, l'extraction des CEATB pose problème du fait de plateau technique obsolète. Dans ces conditions, le recours à d'autres moyens d'extraction s'impose.

Nous rapportons le cas d'un CEATB métallique survenu à guelmim, loin des grandes structures médicales du Maroc, dont l'extraction salvatrice a été réalisée dans un bloc opératoire d'urologie, grâce à l'utilisation d'un urétéroscope.

## Patient et observation

Un enfant de 5 ans, de sexe masculin, sans antécédent particulier, admis aux urgences du 5^ème^ hôpital militaire de Guelmim dans un tableau de détresse respiratoire suite à l'inhalation d'un objet métallique.

**Figure 1:**
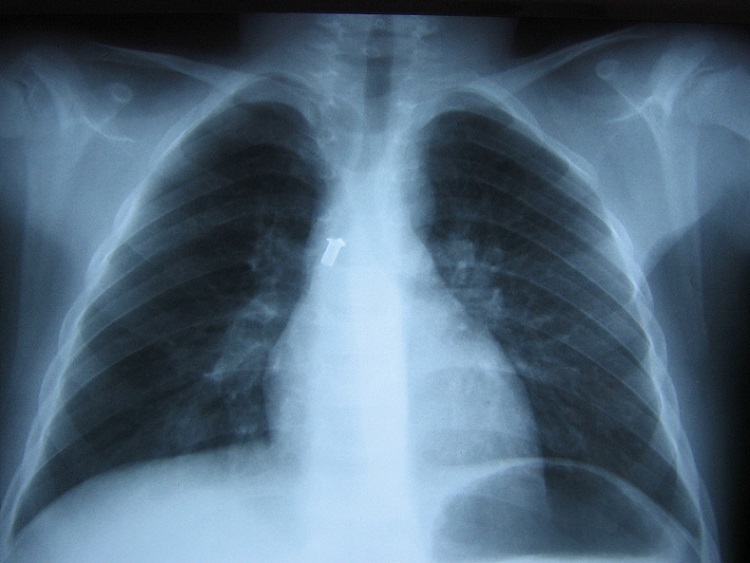
Radiographie pulmonaire objectivant un corps étranger radio opaque en regard de la bronche souche droite

L'examen trouve un enfant obnubilé polypneique avec des signes de lutte respiratoire: tirage intercostal et sus sternal, froideur et sueur des extrémités et début de cyanose péribuccale. L'enfant fut intubé ventilé devant l'aggravation de la symptomatologie malgré l'oxygénothérapie par masque facial. La radiographie standard du thorax (Figure 1) met en évidence un corps étranger métallique intratrachéobronchique.

**Figure 2:**
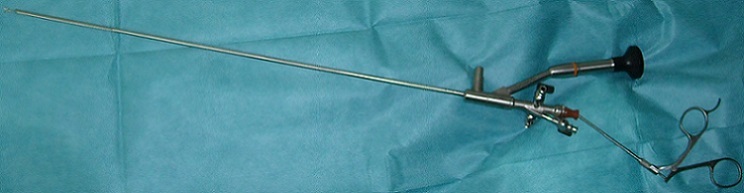
L'urétéroscope utilisé pour l'extraction du corps étranger

Etant donné l'urgence vitale et devant l'absence de structure capable de gérer ce type de problème et notamment un service de réanimation pédiatrique (le centre le plus proche se trouve à plus de 700 Km de Guelmim) et d'un bronchoscope rigide adapté et en concertation avec la famille il a été décidé d'admettre l'enfant au bloc opératoire d'urologie pour tentative d'extraction à l'aide de l'urétéroscope rigide du service (Figure 2).

Dans ce contexte, nous avons concerté toute les expertises chacun dans son domaine associant le médecin urologue, l'otorhinolaryngologiste et l'anesthésiste réanimateur. La préparation et la manipulation du matériel (urétéroscope et pinces) était à la charge des médecins urologue et otolaryngologue et la gestion des voies aériennes au médecin anesthésiste.

Dés la préparation du matériel et l'optimisation de l'oxygénation l'enfant fut extubé pour une première tentative ayant permis la localisation du corps étranger et l'aspiration de l'arbre trachéo bronchique. La deuxième tentative était fructueuse et l'enfant fut reintubé pour ventillation en attendant l’élimination des curares.

**Figure 3:**
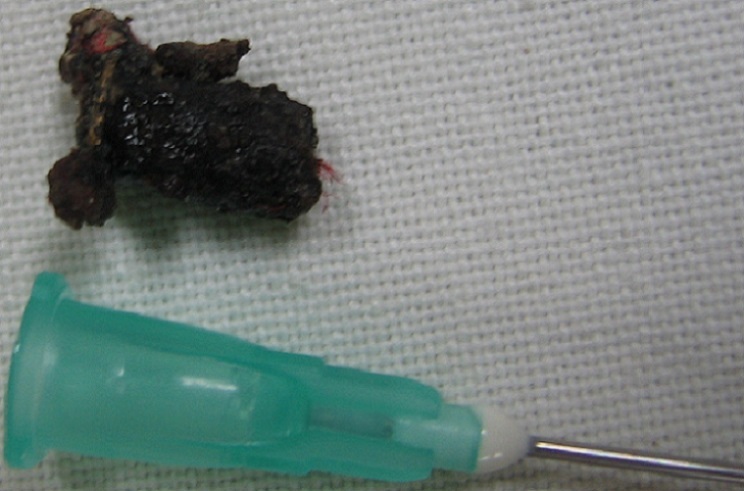
Le corps étranger extrait

L'extraction du corps métallique (Figure 3) est rendu possible grâce à la collaboration des medecins urologue, otolaryngologiste et la supervision de l'anesthésiste réanimateur. L’évolution a été alors très favorable et l'enfant a pu regagner l’école dés le lendemain.

## Discussion

Les CEATB sont suspectés en phase aiguë devant un syndrome de pénétration. Dans ces situations, le diagnostic est fait par la radiographie standard du thorax lorsque les corps étrangers sont radio-opaques. Dans le cas contraire, le CEATB est suspecté sur des arguments cliniques et parfois des anomalies radiologiques indirectes et confirmé par la bronchoscopie souple trachéobronchique [4].

La désobstruction trachéobronchique en urgence par bronchoscopie rigide est parfois la seule solution pour pallier une détresse respiratoire liée à un obstacle des grosses voies aériennes [5], tel est le cas de notre observation. Ailleurs ils peuvent passés inaperçus, ils sont alors responsables d'infections respiratoires répétées, de dilatations de bronche pouvant aller jusqu’à la destruction du parenchyme pulmonaire de l'enfant [4, 6]. Les outils de diagnostic sont la bronchoscopie souple et la radiologie. Le scanner du thorax est plus sensible que la radiographie standard du thorax [7]. Le délai diagnostique de ces CEATB est souvent long.

L'arbre bronchique droit est le siège le plus fréquent des CEATB [6, 8]. Globalement, les CEATB sont majoritairement organiques et singulièrement des végétaux (noix, semences) [8–10). Dans la série de Cataneo et al. les corps radioopaques représentaient 20,7% des cas [11).

La prise en charge thérapeutique des CEATB a été révolutionnée par l'essor de la bronchoscopie souple. Les taux de succès varient entre 61 et 97% selon les séries [10]. La bronchoscopie rigide reste très utilisée surtout dans les services d'otorhinolaryngologie [12]. Ces deux techniques ont réduit considérablement la place de la chirurgie. L'extraction des CEATB est difficile dans les pays pauvres. Le recours à la chirurgie varie entre 6 et 10,4% [9, 10] dans les pays pauvres, pouvant atteindre 13% lorsqu'il s'agit de CEATB de diagnostic tardif [8]. La prise en charge par la bronchoscopie souple, accessoirement la bronchoscopie rigide, est la plus souhaitable [3].

L'extraction des CEATB exige la fibroscopie bronchique pédiatrique. Cela impose un plateau technique qui n'est pas toujours disponible et accessible. Dans notre situation il y avait une urgence vitale et l’évacuation sanitaire vers le centre hospitalier le plus proche est jugée trop risquée.

L'anesthésie générale reste la technique la plus sûre pour garantir le bon déroulement de l'endoscopie trachéobronchique et permettre l'extraction du corps étranger [13]. Il s'agit néanmoins d'une anesthésie à haut risque et les modalités anesthésiques choisies doivent garantir une oxygénation correcte et une profondeur d'anesthésie suffisante, pour permettre la tolérance des manoeuvres endoscopiques, chez un patient à risque d'obstruction complète des voies aériennes. Les facteurs de risque d'hypoxémie peropératoire sont le jeune âge, la durée de l'endoscopie, la nature de corps étranger (végétal), l'existence d'une pneumopathie et le mode ventilatoire (ventilation spontanée) [14].

Le retrait du corps étranger reste une procédure aléatoire et le chirurgien doit être prêt à pratiquer rapidement une trachéotomie ou une cricothyrotomie si l'obstruction partielle se complète brutalement [15]. En cas d'obstruction complète des voies aériennes par enclavement du corps étranger dans la trachée, si celui-ci ne peut être extrait immédiatement, il doit être poussé au-delà de la carène afin de permettre l'oxygénation du patient [16]. En cas de ventilation impossible, tout doit être tenté pour permettre l'extraction rapide du corps étranger. Tous ces points soulignent la nécessité d'une prise en charge anesthésique assurée par un médecin anesthésiste expérimenté en anesthésie pédiatrique [13] et d'une collaboration étroite au sein de l’équipe médico-chirurgicale [17].

Au cours de l'endoscopie au tube rigide, des complications iatrogènes sévères ont été décrites. Leur incidence est de 0,96% [16]: laryngospasme, bronchospasmes, oedèmes laryngés sévères, pneumothorax ou pneumomédiastin, arrêts cardiaques, encéphalopathie anoxo-ischémique, lacérations trachéales et bronchiques [10, 18]. Le risque de décès associé à la réalisation d'une bronchoscopie pour extraction de corps étranger varie entre 0 et 0,94% en fonction des séries [16]. La majorité de ces décès sont consécutifs à un arrêt cardiorespiratoire hypoxique. Dans les autres cas, ils peuvent être consécutifs à une rupture bronchique, à un bronchospasme sévère ou à une complication infectieuse [16]. Compte tenu de cette morbi-mortalité, certains auteurs ont proposé de réserver les indications d'endoscopie au tube rigide en première intention aux situations où il existe un tableau clinique asphyxique, un corps étranger radio-opaque ou des signes très évocateurs de corps étranger inhalés [19, 20]. C’était le cas dans notre observation oû on a opté pour une immobilisation totale grâce à une curarisation optimale. L'extraction, en dépit du manque d'expertise, est rendue possible grâce à la collaboration et la coordination des gestes des différents médecins intervenant.

Dans la littérature on retrouve des cas similaires de tentative d'extraction de CEATB à l'aide de moyen disponible. Il s'agit d'une extraction à l'aide d'une pince à corps étranger, passée à travers la sonde d'intubation orotrachéale et guidée par un amplificateur de brillance dans un bloc opératoire de traumatologie [21]. Cette observation et la notre offrent certainement des alternatives d'urgence variable mais la fibroscopie bronchique rigide reste l'outil de choix et il doit être disponible dans le plateau technique de tous les centres hospitaliers.

## Conclusion

L'extraction des CEATB est handicapée par l'absence de plateau technique adéquat. Ce geste salvateur souligne l'intérêt de l'optimisation des moyens disponibles et la collaboration des différents spécialistes et offre alors une alternative pour l'extraction des CEATB notamment en cas de mise en jeu du pronostic vital.
